# Latest insights on ABO‐incompatible living‐donor renal transplantation

**DOI:** 10.1111/iju.14109

**Published:** 2019-09-14

**Authors:** Junji Uchida, Akihiro Kosoku, Toshihide Naganuma, Tomoaki Tanaka, Tatsuya Nakatani

**Affiliations:** ^1^ Department of Urology Osaka City University Graduate School of Medicine Osaka Japan; ^2^ Department of Urology Suita Municipal Hospital Suita Japan

**Keywords:** ABO incompatibility, desensitization, immunosuppression, renal replacement therapy, renal transplantation

## Abstract

This review summarizes the latest insights on ABO‐incompatible living‐donor renal transplantation. Desensitization protocols and clinical outcomes were investigated, and a comparison was made with kidney‐paired donation, which is not permitted in Japan for ethical reasons. Although renal transplantation is greatly beneficial for most patients with end‐stage kidney disease, many of these patients must remain on dialysis therapy for extended periods due to the scarcity of organs from deceased donors. ABO blood type incompatibility was once believed to be a contraindication to renal transplantation due to the increased risk for antibody‐mediated rejection and early graft loss attributable to isoagglutinins. Recently, pretransplant desensitization strategies, such as removal of isoagglutinins and antibody‐producing cells, have achieved successful outcomes, although it remains unclear whether graft survival and patient morbidity are equivalent to those for ABO‐compatible renal transplantation. The present review suggested that ABO‐incompatible living‐donor renal transplantation might be a favorable radical renal replacement therapy for patients with end‐stage kidney disease.

**Abbreviations & Acronyms**
ABMR: antibody‐mediated rejection
CRT: compatible renal transplantation
DFPP: double filtration plasmapheresis
ESKD: end‐stage kidney disease
EVR: everolimus
HLA: human leukocyte antigen
IA: immunoadsorption
IRT: incompatible renal transplantation
IVIG: intravenous immunoglobulin
KPD: kidney‐paired donation
MMF: mycophenolate mofetil
PE: simple plasma exchange
SePE: selective plasma exchange

## Introduction

Renal transplantation is the preferred renal replacement therapy, because recipients have longer and healthier lives compared with dialysis therapy.^1^, ^2^ Meanwhile, because of the scarcity of deceased donors and constantly growing renal transplant waiting lists, approximately 30% and 90% of all renal transplants in the USA and Japan, respectively, are living‐donor transplants.^3^, ^4^ Strategies have been devised to overcome this shortage in deceased donors, and a patient seeking a renal transplant with only an ABO‐incompatible living donor can now either take part in a kidney exchange program or undergo an ABO‐incompatible transplant.

Researchers who first engaged in the implementation of clinical renal transplantation believed that donors and recipients should be compatible for ABO blood groups.^5^, ^6^, ^7^ ABO blood type incompatibility was considered a contraindication to renal transplantation, because the risk for hyperacute rejection is elevated due to isoagglutinins and early graft loss.^5^ However, desensitization strategies, such as removal of isoagglutinins and antibody‐producing cells, have led to successful ABO‐IRT outcomes.^8^ Patient and graft survival rates have been reported to be equivalent to those of ABO‐CRT,^8^, ^9^ and one‐quarter of living‐donor renal transplantation in Germany and more than one‐third in Japan are ABO‐IRT.^10^, ^11^

However, this type of renal transplantation is still globally uncommon, despite its excellent patient and graft survivals. The potential risks of ABO‐IRT have been studied using data from registry and cohort studies. Although smaller studies on patient and graft outcomes after ABO‐IRT have been comparable to those for ABO‐CRT, larger registry studies have shown conflicting results.^8^, ^12^, ^13^, ^14^

Currently, there are numerous desensitization regimens for ABO‐IRT, but accepted baseline and target isoagglutinin titers, the number of apheresis sessions, apheresis techniques, doses of rituximab, and immunomodulatory strategies remain unestablished. In the present review, we discuss the latest insights on ABO‐IRT.

## Desensitization protocols

Isoagglutinins occur as natural antibodies and are serious immunological obstacles in carrying out ABO‐incompatible organ transplants. If desensitization is not done appropriately, isoagglutinins can cause severe ABMR, and hyperacute rejection can even lead to immediate allograft loss.^15^, ^16^ Recent reports have shown that after a limited period of desensitization after ABO‐IRT, most patients preserve a state of stable long‐term graft function without antigen–antibody interaction, even if recurrence of isoagglutinins is detected. This situation is defined as accommodation.^8^, ^17^ In this respect, ABO‐incompatible transplantation might differ from HLA‐incompatible transplantation, in which persistent or recurrent donor‐specific antibodies often bring about ongoing rejection and chronic tissue injury.^18^

**Figure 1:**
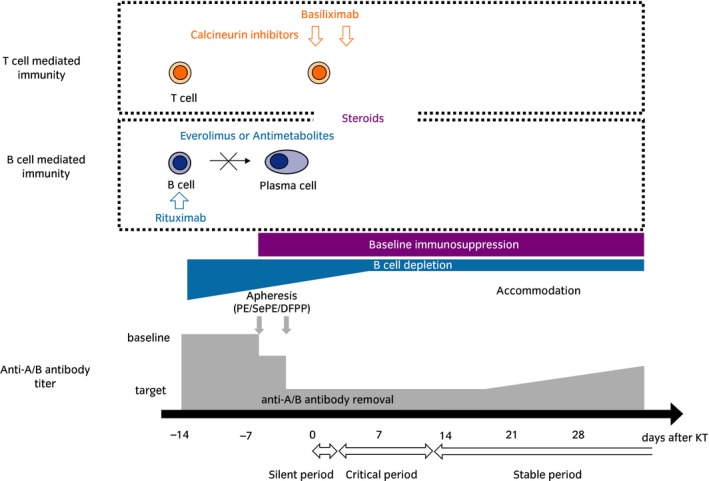
Desensitization strategy for ABO‐incompatible kidney transplantation. Anti‐CD20 therapy is carried out with rituximab administration, and anti‐interleukin‐2 receptor therapy is carried out with basiliximab administration. Administration of EVR or antimetabolites is carried out as B‐cell depletion. To remove anti‐A/B antibodies, apheresis (PE, DFPP, SePE) is carried out.

**Figure 2:**
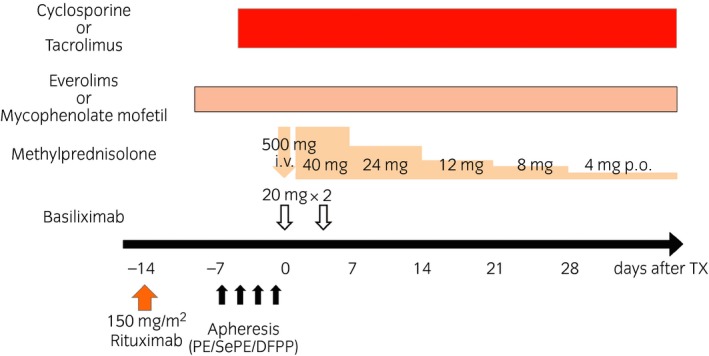
Standard desensitization protocol for ABO‐incompatible kidney transplantation at Osaka City University Hospital. Tx, kidney transplantation.

Desensitization protocols allowing for successful ABO blood type‐IRT had not been established until recently.^19^, ^20^ Key elements of desensitization regimens for ABO‐IRT are the application of apheresis for isoagglutinin removal, pre‐emptive modulation of B‐cell immunity and pharmacotherapy as maintenance immunosuppression (Fig. 1). ABO‐IRT is immunologically high risk, and desensitization regimens should be modified based on recipient age, rebound of isoagglutinins and baseline isoagglutinin titers.^12^ The standard immunosuppressive therapy for ABO‐IRT at the Department of Urology, Osaka City University Graduate School of Medicine, Osaka, Japan is shown in Figure 2, and modified desensitization protocols might be important in preventing infections and avoiding rejections.

### B‐cell immunomodulation

Initially, splenectomy was carried out for inhibiting isoagglutinin production in ABO‐IRT, because a large pool of antibody‐secreting B cells and plasma cells are contained in the spleen.^21^ However, in the past decade, due to the surgical risk, as well as an increased risk of sepsis, splenectomy has been replaced with the administration of rituximab, an anti‐CD20 antibody that induces B‐cell depletion in the peripheral blood, although the optimal dose and frequency of administration remain unknown.^20^, ^22^ A single dose or two doses of rituximab are administered for B‐cell immunomodulation at major transplant centers. A single dose of rituximab effectively removes peripheral B cells, but might not decrease B‐cell counts in secondary lymphoid organs.^23^

There have been few reports on ABO‐IRT desensitization protocols for patients with elevated isoagglutinin titers, and the regimen for these patients has not yet been established.^24^ Meanwhile, baseline isoagglutinin titers can forecast early ABMR.^25^ We reported that using a desensitization protocol consisting of both rituximab administration and splenectomy in two highly HLA‐sensitized patients and six patients with elevated (>512‐fold dilution) isoagglutinin titers, renal transplantation was successfully carried out in seven of the eight patients without grave complications.^26^ In another report, three doses of rituximab administration and MMF administration for 210 days were successfully used in a patient with elevated isoagglutinin titer (4096‐fold dilution) and high refractory isoagglutinins.^27^

There is controversy surrounding the effect of rituximab on de novo formation of donor‐specific antigen.^28^ A recent randomized control study reported that transplant outcomes of ABO‐CRT did not benefit from rituximab induction.^29^ In contrast, Clatworthy *et al*. reported that there was a relationship between B‐cell‐related cytokine release due to rituximab administration and a higher incidence of acute rejection in patients treated with rituximab.^30^ However, another report showed that recipients in whom rituximab was administered as an ABO‐IRT desensitization protocol did not appear to be at an increased risk for acute cellular rejection.^31^ Any cytokine storm would have been resolved before transplantation by preoperative plasma exchange and corticosteroid therapy, and no increase in the risk of rejection would be expected.^30^

The administration of IVIG is a widely used component of immunomodulatory strategies that prevents isoagglutinin rebound in the early stage after ABO‐IRT. The interaction of the constant fragments of IVIG with Fc receptors of phagocytes and B cells suppresses further differentiation and T‐cell stimulation, whereas the variable fragments of IVIG inhibit autoantibodies from binding to their specific receptors.^32^ Furthermore, IVIG can lead to the secretion of anti‐inflammatory cytokines and act as “blocking” antibodies in cross‐match tests *in vitro*, as well as in clinical observation of immediately decreased HLA antibodies after infusion.^33^ However, a potential disadvantage of IVIG therapy is that ABO antibodies are contained in the available preparations, which can induce a temporary increase in titers.^34^

### Anti‐A/B antibody removal

Pretransplant apheresis for the removal of isoagglutinins until the target titers are reached is fundamental for most ABO‐IRT desensitization protocols, although the optimal target titers remain to be  unestablished.^9^ Apheresis sessions are scheduled based on baseline isoagglutinin titers. Antibody removal might be imperative before transplantation to prevent ABMR, but might not be necessary after transplantation, when isoagglutinins frequently recur without causing detectable harm.^8^, ^17^ However, some protocols include post‐transplant apheresis for cases with early antibody rebound.^25^, ^35^

There are several available apheresis techniques for the removal of isoagglutinins, including DFPP, PE and antigen‐specific IA. In DFPP, substantial amounts of macromolecular coagulation factors, in particular fibrinogen or factor XIII, can be lost, increasing the risk of bleeding complications.^36^ With PE, side‐effects such as allergic reactions to fresh frozen plasma have been reported to result in suspension of treatment.^37^ IA is a selective strategy to remove antibodies. Antigen‐specific IA eliminates isoagglutinins highly efficiently without major losses of essential plasma components.^38^ However, it is an expensive treatment, with columns costing approximately €3000 each, and the application of this column for therapeutic apheresis is not approved in Japan.^39^

In SePE, which is a new PE modality using a smaller pore size membrane plasma separator compared with conventional plasma separators, small and medium‐sized molecules are eliminated without removing larger substances, such as coagulation factors.^40^ Recently, SePE is being applied to decrease isoagglutinin titers in pretransplant desensitization for ABO‐IRT. Because SePE is less efficient in the removal of isoagglutinin titers compared with conventional methods, SePE alone should be only used in patients with low titers, and a combination of SePE with conventional methods should be used in patients with high titers.^41^

Previous reports showed that patients with decreased isoagglutinin titers can undergo ABO‐IRT based on standard desensitization not only without B‐cell depletion, such as rituximab administration, but also without isoagglutinin removal.^9^, ^42^, ^43^ Therefore, these desensitization protocols could be simplified in the future. However, when ABO‐IRT was carried out in patients with decreased isoagglutinin titers without removing isoagglutinins, they were reported to have acute ABMR, resulting in graft loss.^44^

## Outcomes of ABO‐incompatible living‐donor renal transplantation

### Patient and graft survivals

The latest systematic reviews and meta‐analyses based on transplantation data for ABO‐CRT and ABO‐IRT in studies from the USA, Europe, Asia and Australia showed that graft loss and death within the first 3 years of transplantation were more often observed in ABO‐IRT compared with ABO‐CRT. Equivalent survival rates and organ functions were only seen after 5 years post‐transplant. Desensitization with rituximab brought about a comparable death‐censored graft survival between the two groups within the first year, and excess mortality was observed in ABO‐IRT only within the first 3 years. Death‐censored graft survival was equivalent between the ABO‐IRT and ABO‐CRT groups at 1 year if the initial desensitization protocol included rituximab, and it was worse in the ABO‐IRT group compared with the ABO‐CRT group if the initial desensitization protocol did not include rituximab. This was also true for death‐censored graft survival at 3 years. Studies on graft survival after 5 years did not show a significant difference between the two groups, when analyzed according to whether or not they had undergone rituximab treatment.^13^ Another meta‐analysis study showed that graft survival was lower in ABO‐incompatible recipients compared with ABO‐compatible recipients. The risk ratio for 1 year was lower in ABO‐incompatible patients than in ABO‐compatible patients. Graft survival remained lower in patients with ABO‐IRT at 3 years (92% *vs* 94%, *P* = 0.04). The 1‐year survival was also lower in ABO‐incompatible recipients (98% *vs* 99%, *P* = 0.03).^45^

**Figure 3:**
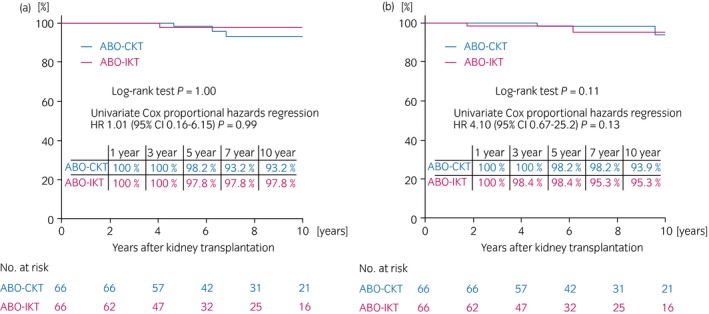
(a) Patient and (b) graft survival rate of ABO‐incompatible kidney transplantation and ABO‐compatible kidney transplantation after propensity matching (updated version of Figure 4 from Kosoku *et al*.^12^ with permission).

Since 1989, more than 2000 ABO‐IRT have been carried out in Japan. A 2006 Japanese registry analysis showed that ABO‐IRT survival rates were acceptable, but still lower than those of ABO‐CRT.^21^ A follow‐up analysis of Japanese recipients receiving a graft from 2001 to 2010 showed excellent long‐term ABO‐IRT outcomes, possibly due to improvements in MMF and/or rituximab.^21^ One single‐center study in Japan showed that graft survival was significantly lower for ABO‐IRT than for ABO‐CRT, and that there were significantly more frequent graft losses due to infection in ABO‐IRT than in ABO‐CRT.^46^ In another Japanese single‐center study, graft survival for ABO‐IRT was almost the same over the past decade compared with that for ABO‐CRT.^11^ Previously, we showed that both patient and death‐censored graft survival rates were not significantly different between the ABO‐IRT and ABO‐CRT groups.^12^ Figure 3 provides the updated results of our outcomes until March 2019. However, the differences in transplant outcomes between these Japanese single‐center studies and meta‐analysis studies might be due to the small number of patients and/or the differences in patients’ background, such as desensitization protocols, antibody titers and management system after transplantation.

A USA registry analysis comparing the outcomes of ABO‐IRT and ABO‐CRT between 1995 and 2015 showed a high rate of early graft loss after ABO‐IRT, but no change in long‐term patient survival. That study also showed no significant change between low‐volume versus high‐volume centers.^14^

Although these data clearly showed that ABO‐IRT has achieved excellent outcomes in recent years, it remains unclear whether graft survival and patient morbidity for ABO‐IRT are comparable to those for ABO‐CRT. Even with advanced desensitization protocols that include rituximab, patient mortality might be higher in the early stages after ABO‐IRT than after ABO‐CRT.

Renal transplants from poor HLA matching or HLA‐incompatible donors are considered to be immunologically high‐risk, as with ABO‐IRT. Although graft survival rates in poor HLA‐matched recipients used to be significantly lower than those in fully HLA‐matched recipients,^47^ currently, there are no significant differences in graft survival rates according to the number of HLA mismatches, owing to the development in immunosuppressants.^48^, ^49^ The presence of donor‐specific anti‐HLA antibodies increases the risk for ABMR and graft failure.^50^, ^51^ However, a desensitization therapy for highly HLA‐sensitized patients has not been established yet. HLA‐IRT also has lower graft survival rates compared with ABO‐IRT.^52^ Nevertheless, it has been reported that patients who receive HLA‐incompatible kidney transplants have a higher survival rate than those who remain on dialysis therapy.^53^

### Rejection

Systematic reviews and meta‐analyses based on transplantation data for ABO‐CRT and ABO‐IRT in studies from the USA, Europe, Asia and Australia showed no significant difference between ABO‐CRT and ABO‐IRT in overall, borderline or TMR. In contrast, there was a higher percentage of patients with ABMR after ABO‐IRT than after ABO‐CRT.^13^ However, the risk of ABMR after ABO‐IRT was similar to that after ABO‐CRT at 5 years after transplantation, when a rituximab desensitization protocol was initially used.^13^ Although rituximab does not seem to be effective in decreasing the concentration of preformed HLA antibodies or preventing the development of de novo HLA antibodies, the risk of humoral rejection may be reduced. Another review showed that biopsy‐proven acute rejection, especially ABMR, was more prevalent in ABO‐IRT.^45^

In a Japanese single‐center study, Okada *et al*. also showed that the risk of acute ABMR was significantly higher in ABO‐IRT than in ABO‐CRT, although the prevalence of acute TMR and chronic ABMR was not significantly different between the two groups.^46^ However, another single‐center study from Japan showed that the incidence of rejection episodes, such as acute cellular rejection, steroid‐resistant acute cellular rejection and ABMR, might be comparable between the ABO‐IRT group and the ABO‐CRT group.^12^ Differences in the rate of ABMR between these Japanese single‐center studies and meta‐analysis studies might be due to the small number of patients and/or the differences in patients’ background, such as desensitization protocols, antibody titers and management system, after transplantation.

## Complications

### Infectious complications

Desensitization in combination with immunosuppressants can lead to a state of over immunosuppression, causing infections. Previous articles have shown that there are conflicting results about infectious complications after ABO‐IRT. A recent systematic review and meta‐analysis showed a higher proportion of patients with sepsis after ABO‐IRT than after ABO‐CRT, although the risk of urinary tract infection, cytomegalovirus infection, BK polyomavirus infection and *Pneumocystis jirovecii* pneumonia was not significantly different.^13^ Furthermore, there was a significantly higher risk of sepsis and cytomegalovirus infection after ABO‐IRT than after ABO‐CRT in patients who received non‐rituximab‐based desensitization protocols, but there was no difference between the treatment groups among those who received rituximab‐based desensitization protocols.^13^ Rituximab induction was considered to be relatively safe and not associated with infections in a large, randomized trial of kidney transplant recipients.^54^ However, in the Collaborplant Transplant Study registry, rituximab induction for ABO‐IRT was associated with infectious complications compared with no induction therapy, although death‐censored graft survival was better.^22^

A different meta‐analysis study showed that there was a higher frequency of severe non‐viral infections in ABO‐IRT than in ABO‐CRT. Cytomegalovirus viremia was slightly more commonly observed in ABO‐incompatible patients compared with ABO‐compatible patients.^47^ However, there is no definitive evidence of high frequency of infection after ABO‐IRT compared with ABO‐CRT.^25^, ^55^

In a Japanese study, the frequency of infectious adverse events was not significantly different between the ABO‐incompatible and ‐compatible groups in the past decade (from 2005 to 2013).^11^ A single low‐volume center study carried out in Japan also showed no significant change in the prevalence of infectious adverse events between the ABO‐incompatible and ‐compatible groups, and no critical infectious complications were observed during the observation duration (median 6.04 years) in the ABO‐incompatible group.^12^ However, another Japanese study reported that there was a higher cumulative incidence of infection in ABO‐IRT than in ABO‐CRT, implying that desensitization can elevate infection risks soon after transplantation.^46^

### Bleeding

Apheresis to deplete isoagglutinins can cause bleeding complications, which might be explained by coagulation factors, including fibrinogen.^56^, ^57^ A single PE reduces the amount of coagulation factors by approximately 60%, and DFPP and IA also significantly reduce the amount of coagulation factors.^38^, ^58^, ^59^ Fibrinogen has the maximum concentration amongst all coagulation factors. A fibrinogen level of 100 mg/dL is required to maintain hemostasis, and replacement of fibrinogen is recommended when <100 mg/dL at transplantation.^60^ Higher bleeding rates have been shown after ABO‐IRT than after ABO‐CRT, possibly because of changes in the coagulation system after plasmapheresis, high volume of exchange or IA.^13^ de Weerd *et al*. showed that bleeding events occurred almost twice as often in ABO‐incompatible patients versus ABO‐compatible patients, both with IA and with plasmapheresis.^45^

### Late‐onset neutropenia

Some reports have shown that rituximab administration leads to late‐onset neutropenia after ABO‐IRT.^31^, ^61^, ^62^, ^63^ A previous report showed that late‐onset neutropenia after rituximab administration in ABO‐IRT recipients was related to the increase in serum B‐cell activating factor.^32^ Our recent reports showed that late‐onset neutropenia was related to acute cellular rejection in ABO‐IRT recipients undergoing rituximab administration.^61^, ^62^ Late‐onset neutropenia after rituximab administration might therefore be associated with B‐cell‐related cytokine.

## ABO‐IRT in pediatrics

Contrary to ABO‐IRT in adults, there are few reports regarding pediatric ABO‐IRT. A Japanese multicenter study of pediatric kidney transplantation showed that outcomes, such as death and graft loss rates, for ABO‐IRT were similar to those for ABO‐CRT.^64^

## ABO‐incompatible transplantation of other organs

Because there is the “safety net” of dialysis as rescue treatment for patients whose renal grafts fail, renal transplant recipients can take higher risks than other organ transplant recipients. In addition, ABO‐incompatible transplantation in other organs has been addressed based on the success of ABO‐IRT. Numerous reports have been published on successful ABO‐incompatible transplantation of various organs, including the lung, liver and heart, in both children and adults.^65^, ^66^, ^67^, ^68^, ^69^, ^70^

In lung transplantation, there is no comparative data between ABO‐incompatible and ‐compatible transplants, because there were few cases of ABO‐incompatible lung transplantation.^65^, ^66^ In liver transplantation, there are no significant differences in patient and graft survival rates between ABO‐incompatible (*n* = 235) and ABO‐compatible (*n* = 470) transplants in a propensity score‐matched cohort. The 5‐year graft and patient survival rates of the ABO‐incompatible group were 89.9% and 92.3%, respectively, which were comparable to those of the ABO‐compatible group (91.2% and 91.4%).^67^ In heart transplantation, patients and/or graft survival rates 7 years after ABO‐incompatible (*n* = 35) and ABO‐compatible (*n* = 45) were both 74%, and not significantly different.^69^ There were no significant differences in medium‐term outcomes between ABO‐incompatible and ABO‐compatible transplantation, although serious concerns persist about ABMR.^67^, ^69^

## Mammalian target of rapamycin inhibitor for ABO‐incompatible kidney transplantation

Most immunosuppressive protocols for ABO‐IRT consist of rituximab induction, apheresis or immune absorption, and maintenance immunosuppression based on tacrolimus, MMF and steroids. Koch *et al*. reported the results of 25 patients with ABO‐IRT on a de novo mammalian target of rapamycin inhibitor‐based immunosuppression regimen to prevent allograft rejection without increasing the risk of viral infection, and showed that this regimen was feasible without severe surgical or immunological complications and a low rate of viral infection.^71^

Safe switch from MMF with standard‐dose calcineurin inhibitor to EVR with low‐dose calcineurin inhibitor in 16 stable ABO‐IRT recipients at maintenance duration was reported.^72^ However, treatment with EVR was discontinued due to adverse effects in 47.1% of the patients within 1 year of conversion. In addition, the switch to EVR did not lead to acute rejection or C4d deposition at 3 and 12 months after conversion in ABO‐IRT recipients in whom EVR was continued or discontinued within 1 year of conversion.^73^

In another study, seven stable ABO‐IRT recipients who were switched from MMF to EVR at a late post‐transplant stage due to BK virus replication were compared with a reference group of 14 ABO‐IRT patients given standard tacrolimus and MMF.^74^ As a result, conversion from MMF to EVR decreased BK replication in five patients. That study showed that conversion to EVR was beneficial for ABO‐IRT recipients with BK viral infection.

## Kidney‐paired donation

The concept of ABO‐IRT is to overcome incompatibility by removing the ABO‐incompatibility barrier, whereas that of KPD is to do so by avoiding the barrier. Many KPD programs have been developed and range from simple two‐way exchanges to long, so‐called domino chains with bridging donors requiring sophisticated matching algorithms and software.^75^ The first KPD was carried out in Korea in 1991,^76^ and currently, it is being carried out in many other countries including the Netherlands, the USA, Canada, Australia, the UK, Turkey and India.

A previous report showed that the waiting period for all registrants was >1 year at a mean of 747 ± 322 days.^77^ Clinical outcomes of KPD might be poorer than those of ABO‐IRT because of longer waiting periods of >6 months, but large‐scale clinical trials comparing ABO‐IRT with KPD are necessary.^78^ In addition, KPD has some limitations, such as refusal of the intended donor to donate in exchange, blood group imbalance (disadvantage for blood type O recipients), disparity in quality of organs, geographical distance and legal barrier.^75^, ^79^

Compared with ABO‐IRT, the advantages of KPD are cost‐effectiveness, low immunological risk and avoidance of desensitization complications, including infection and bleeding.^11^, ^12^ Because ABO‐IRT has overcome the ABO‐incompatibility barrier in Japan, where KPD is not allowed due to ethical reasons, such a program might not be necessary.

## Pros of ABO‐incompatible kidney transplantation

*In vitro* experiments have shown that isoagglutinin ligation‐induced resistance to HLA antibody‐mediated, complement‐dependent cytotoxicity through the upregulation of complement regulatory proteins and downregulation of HLA‐DR expression protects against antibody‐mediated injury. A recent clinical study showed that the incidence of DR‐associated de novo donor‐specific antigen and biopsy‐proven chronic ABMR was lower in ABO‐IRT than in ABO‐CRT.^80^ ABO‐incompatibility might lower the production of DR‐associated de novo donor‐specific antigen, possibly decreasing the incidence of chronic ABMR.

## Challenging cases of ABO‐incompatible living‐donor renal transplantation

As mentioned above, ABO‐IRT has become a favorable renal replacement therapy for patients with ESKD. However, few reports have been made on higher risk ABO‐IRT, such as for elderly patients, patients with diabetic kidney disease and second transplants.

### Elderly ABO‐incompatible living‐donor renal transplantation

A recent study of 17 patients aged ≥60 years who underwent ABO‐IRT achieved both overall patient and graft survival rates of 100%, 100% and 83.3% at 1, 3 and 5 years after their transplants, respectively.^81^

### ABO‐IRT in patients with diabetic kidney disease

Uchida *et al*. showed that among 14 patients with diabetic kidney disease who received ABO‐incompatible grafts, two (14.3%) developed biopsy‐proven acute cellular rejection during the follow‐up period.^82^ Patient survival rates were 100%, 89.9% and 89.9% at post‐transplant 1, 3 and 5 years, respectively, and the death‐censored graft survival rate at 5 years was 100%.

### ABO‐IRT as a second transplant

A recent study reported three successful cases of patients who underwent ABO‐incompatible living‐donor kidney transplantation as a second transplant.^83^

These results showed that ABO‐IRT might now be an acceptable treatment for challenging cases, such as elderly ESKD patients, ESKD patients due to diabetic kidney disease and patients who require a second renal replacement therapy after their initial graft failure.

## Conclusion

In conclusion, the present review of ABO‐IRT has shown very good outcomes, although it remains unclear whether graft survival and patient morbidity for ABO‐IRT are comparable with those for ABO‐CRT. ABO‐IRT might be a favorable radical renal replacement therapy for ESKD patients.

## Conflict of interest

None declared.
